# Association between kidney function and biological age: a China Health and Retirement Longitudinal Study

**DOI:** 10.3389/fpubh.2023.1259074

**Published:** 2023-12-18

**Authors:** Shanshan Peng, Rui Xu, Kai Wei, Na Liu, Yuan Lv, Yong Lin

**Affiliations:** ^1^Department of Laboratory Medicine, Huashan Hospital, Fudan University, Shanghai, China; ^2^Health Management Centre, Huashan Hospital, Fudan University, Shanghai, China; ^3^Department of Rheumatology, Huashan Hospital, Fudan University, Shanghai, China

**Keywords:** kidney, aging, biological age, CHARLS, eGFR

## Abstract

**Introduction:**

The chronological age (CA) cannot precisely reflect the health status. Our study aimed to establish a model of kidney biological age to evaluate kidney function more elaborately.

**Methods:**

The modeling group was used to establish the model, consisting of 1,303 respondents of the China Health and Retirement Longitudinal Study (CHARLS). The biological age of the kidney (BA) was constructed by principal component analysis (PCA) and Klemera and Doubal’s method (KDM) with the 1,303 health respondents.

**Results:**

PCA was chosen as the best method for our research step by step. The test group was used to apply the model. (a) BA of the kidney can distinguish respondents with from without kidney disease. (b) BA of the kidney was significantly different in various levels of kidney function. The BA of the eGFR <60 group and 60 ≤ eGFR <90 group were older than GFR ≥90 group. (c) The group with younger BA of kidney at baseline had a lower risk of kidney function decreased. (d) The risk of decreased kidney function caused by increasing BA every additional year is higher than *CA.*

**Discussion:**

The BA of the kidney is a parameter negatively correlated with decreased kidney function and fills the blank of evaluation among people in the middle of heathy and kidney diseases.

## 1 Introduction

Genes, environment, lifestyle, and other factors influence longevity. There is a delicate balance between aging and diseases, which has become a focus of aging research. The primary aging mechanism includes oxidative stress, inflammation, disturbances in sympathetic-vagal balance, and many others (1). However, chronological age no longer accurately reflects a person’s health. For every one-year increase in the calculated biological age and chronological age difference, the hazard ratio for mortality increased by 1.6% (1.5% in men and 2.0% in women,) as well as for hypertension, diabetes mellitus, heart disease, stroke, and cancer incidence by 2.5, 4.2, 1.3, 1.6, and 0.4%, respectively (2). Therefore, accurately quantifying the aging rate is not only important for evaluating the efficacy of aging interventions, but it also sheds light on the aging process itself (3). The earliest research on biological age dates back to at least the 1960s when scientists sought meaningful biomarkers to quantify the biological age of individuals exposed to the radiation from Hiroshima (4). Researchers have investigated various types of biological age, from the whole body to molecular levels (5–9). They have also investigated different biological age estimation methods, including multiple linear regression (MLR), Hochschild’s method, the principal component analysis (PCA), the Klemera and Doubal’s method (KDM) (8, 10–12).

Different organs have different aging patterns and orders due to their unique physiological structures; therefore, biological age should be calculated according to organs (13). The aging of organs is a gradual process. The kidney undergoes different structural changes as it ages, including glomerulosclerosis, tubular atrophy, and a reduction in cortical volume (14). These changes result in functional degeneration, including a decrease in eGFR, renal blood flow, renal vascular permeability, reabsorption, and urine concentration (15). As China’s population ages, the high cost of treating age-related diseases exceeds the affordability of the country’s limited medical resources. In addition, morbidity and mortality increased as the incidence of kidney injury increased worldwide. Early patient identification is important to provide prompt intervention and improve prognosis (16). Therefore, monitoring the health status is a way to improve the life quality of older adults and reduce healthcare costs. In our study, healthy kidney people were used to model a biological kidney age for evaluating kidney function and aging among the population. The modeling people are from the China Health and Retirement Longitudinal Survey (CHARLS) database and the modeling methods are PCA and KDM.

## 2 Materials and methods

### 2.1 Data

CHARLS is a longitudinal database created by the National School for Development at Peking University that represents families and individuals aged 45 and above in China. The questionnaire includes demographic information, finances, health status, physical measurements, health insurance and retirement status. The pre-survey investigated 17,708 individuals in 28 provinces in 2011 (Wave 1), and follow-up waves were conducted in 2013 (Wave 2) and 2015 (Wave 3). Additional information is available on the CHARLS website^1^ (17). We have registered on the CHARLS website and obtained permission from Peking University. Our study was carried out following approved guidelines and includes data from 2011 and 2015 from the participants who provided written informed consent. The personal information, such as ID and address, was removed from the dataset and coded as a series of numbers. The study protocol was approved by the Ethical Review Committee of Peking University (IRB00001052-11015).

### 2.2 Study population

The study population included individuals with completed data of demographic information, laboratory tests, and health status. Before the baseline examination, respondents diagnosed with kidney-related diseases, including hypertension, diabetes mellitus, and kidney diseases were excluded. The subjects were also excluded due to unqualified laboratory tests and physical examinations, estimated Glomerular Filtration Rate < 60 mL/min/1.73m^2^, fasting blood-glucose >7.0 mmol/L, and non-fasting blood-glucose >11.1 mmol/L, glycated hemoglobin ≥6.5%, systolic blood pressure ≥ 140 mmHg, and diastolic blood pressure ≥ 90 mmHg.

### 2.3 Blood samples and physical examinations collection and analysis

A total of 9 blood test results of the CHARLS were used to evaluate health and develop the BA model of the kidney. Blood tests included estimated glomerular filtration rate (eGFR), fasting blood glucose (glu), non-fasting blood glucose (uglu), glycated hemoglobin (HbA1c), uric acid (ua), creatinine (crea), urea nitrogen (bun), and cystatin C (cysc). Physical examinations included blood pressure. Medically trained personnel from the China CDC collected venous blood from each respondent and transported at 4°C to local CDC laboratories or township-level hospitals near the study sites for a complete blood count test. The cryovials were then frozen at −20°C and transported within two weeks to the Chinese CDC in Beijing, where they were placed in a deep freezer and stored at −80°C until further analysis at the laboratory of Capital Medical University. eGFR was calculated using the CKD Epidemiological Collaboration equation and the chronic kidney disease epidemiology collaboration equations in Asian (CKD-EPI-Asian) (18). eGFR (mL/min/1.73 m^2^) = 151 × (Scr/0.7)^−0.328^ × (0.993)^Age^, (if female and Scr ≤ 0.7). eGFR (mL/min/1.73 m^2^) = 151 × (Scr/0.7)^−1.210^ × (0.993)^Age^, (if female and Scr>0.7). eGFR (mL/min/1.73 m^2^) = 149 × (Scr/0.9)^−0.415^ × (0.993)^Age^, (if male and Scr ≤ 0.9). eGFR (mL/min/1.73 m^2^) = 149 × (Scr/0.9)^−1.210^ × (0.993)^Age^, (if male and Scr>0.9). Blood glucose levels in CHARLS were converted from mg/dL into mmol/L by dividing with 18. The systolic and diastolic pressure was measured three times at 45-s intervals. We selected the highest values as the final systolic and diastolic pressure.

### 2.4 Demographic collection and analysis

Age and gender were major determinants of biological age. As a result, we collected the age, gender, and ID of each respondent from the demographic information in the CHARLS database. In addition, kidney diseases, diabetes, and hypertension were included in the demographic data used to determine whether the kidneys are healthy or unhealthy.

### 2.5 Principal component analysis

There were five steps to develop a biological age formula: correlation analysis, stability analysis, redundancy analysis, principal component analysis (PCA), and formula construction. Identifying biomarkers related to chronological age for the next steps was essential. In our study, correlation coefficient |*r*| > 0.10 was an inclusion criterion. The stability analysis was only used in longitude studies to determine whether biomarkers were stable in two continuous studies. The selected biomarkers were then entered in the next step. The redundancy analysis identifies biomarkers from the same major organ or body part. In other words, this analysis eliminated the overlapping biomarkers, leaving the remaining ones uncorrelated (19). The core of the PCA is dimensions reduction and selection of eigenvalue >1.0 as principal components. When principal components are validated, we typically construct two formulas with and without *CA.* The formula with CA investigated the relationship between principal components and *CA.* The formula without CA investigated whether principal components maintain the relationship without *CA.* The biological age score (*BAS*) is calculated by Equation (1)


(1) $$\begin{array}{c} BAS=a\times \frac{X_{1}-mean_{1}}{SD_{1}}+b\times \frac{X_{2}-mean_{2}}{SD_{2}}\\ +\cdots +n\times \frac{X_{n}-mean_{n}}{SD_{n}} \end{array}$$

Where *n* is the score coefficient, *X_n_* represents the marker, and the mean is the average value of *X_n_*. *SD_n_* is the standard deviation of *X_n_*. Some researchers are used to converting BAS into BA using the T-score method [Equation (2)]. Because BAS has no unit, it cannot be directly compared with CA, namely:


(2) $$BA=BAS\times BA_{SD}+CA_{mean}$$

Where *BA_SD_* is the standard deviation of BA, and *CA_mean_* is the average value of *CA.* To solve the problem of edge data distortion, some researchers have corrected the formula [Equation (3)] is


(3) CorrectedBA=BA+Z

(4) $$Z=\left(y_{i}-y\right)\left(1-b\right)$$

Where *y_i_* is the individual’s CA, *y* is *CA_mean_*, and *b* is the linear correlation coefficient of BA, CA and *Z* is calculated by Equation (4) (10).

### 2.6 Klemera and Doubal’s method

Klemera and Doubal’s method was proposed in research titled “A new approach to the concept and computation of biological age” by Klemera and Doubal in 2006 (20). It is a new mathematical algorithm for calculating biological age confirmed by other papers. The BA estimates are determined by minimizing the distance between *m* regression lines and m biomarker points in an *m-dimensional* space containing all biomarkers. In this article, the authors used computer-generated simulations to validate the method. They defined BA as equivalent to CA and added variables to improve precision. They presented two alternative methods for calculating the optimum estimates of BA, Equations (5) and (6), in which the latter method utilizes CA in the final equation—and using simulations was shown to be superior:


(5) $$BA_{E}=\frac{\sum_{j=1}^{m}\left(x_{j}-q_{j}\right)\left(\frac{k_{j}}{s_{j}^{2}}\right)}{\sum_{j=1}^{m}{\left(\frac{k_{j}}{s_{j}}\right)}^{2}}$$

(6) $$BA_{EC}=\frac{\sum_{j=1}^{m}\left(x_{j}-q_{j}\right)\frac{k_{j}}{s_{j}^{2}}+\frac{CA}{s_{BA}^{2}}}{\sum_{j=1}^{m}{\left(\frac{k_{j}}{s_{j}}\right)}^{2}+\frac{1}{s_{BA}^{2}}}$$

where *j* is one of the *m* biomarkers, *q, k*, and *s* are the intercept, slope, and root mean square error of regression of chronological age on *j*, a is chronological age, and $s_{BA}^{2}$ is a scaling factor equal to the square root of the variance in chronological age explained by the m biomarkers in the CHARLS. Intermediate variables involved in Equations (7) and (8) are calculated as follows.


(7) $$r_{char}=\frac{\sum_{j=1}^{m}\frac{r_{j}^{2}}{\sqrt{1-r_{j}^{2}}}}{\sum_{j=1}^{m}\frac{r_{j}}{\sqrt{1-r_{j}^{2}}}}$$

(8) $$\begin{array}{c} s_{BA}^{2}=\left(\frac{\sum_{j=1}^{n}{\left(\left(BA_{Ei}-CA_{i}\right)-\sum_{i=1}^{n}\frac{BA_{Ei}-CA_{i}}{n}\right)}^{2}}{n}\right)\\ -\left(\frac{1-r_{char}^{2}}{r_{char}^{2}}\right)\times \left(\frac{\left(CA_{\max}-CA_{\min}\right)}{12m}\right) \end{array}$$

Where *r_j_^2^* is used to calculate the characteristic correlation coefficient from Equation (7), it refers to the variance explained by regression *CA* on *m* biomarkers. Finally, following the assumption made by Klemera and Doubal, $s_{BA}^{2}$ was transformed so that $\;s_{BA}$ maintained the same mean but was now linearly increasing with age, with a difference of four between subjects at $CA_{\max}$ and $CA_{\min}$ .

### 2.7 Statistics analysis

The characteristics of respondents were described and compared according to the different biological age estimated methods. Continuous variables were presented as mean (standard deviation, SD) for normal distribution and median (interquartile range, IQR) for skewed distribution. Categorical variables were presented as a number. Mann–Whitney and Kruskal-Wallis tests were used to compare the difference between two groups and more than two groups, respectively. Multinomial logistic regression analysis was used to calculate odds ratios (OR) and 95% confidence interval (CI) for the biological age of different estimated methods and different groups. All statistical analyses were performed using SPSS version 17.0 (SPSS Inc., Chicago, IL, USA), Stata version 13.0 (Stata Corporation, College Station, TX, USA), GraphPad Prism 9.5 (GraphPad Software, San Diego, CA, USA), and the statistical software package R, version 4.1.0.^2^

## 3 Results

### 3.1 The inclusion criteria of respondents in different groups

**Figure 1:**
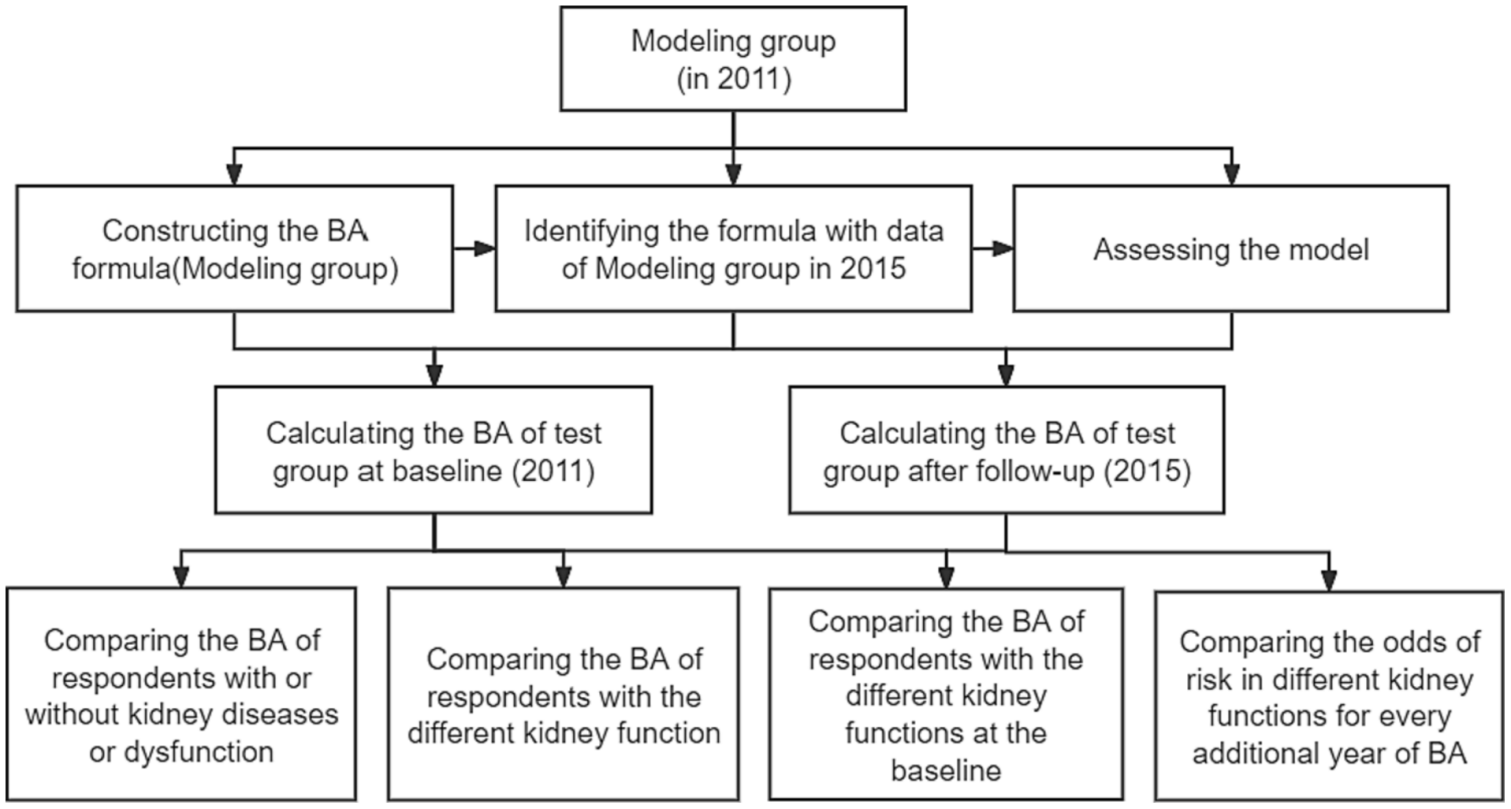
The flowchart of the present study.

Our study utilized two biological age-estimated methods, PCA and KDM. The modeling group included respondents with healthy kidney function from both 2011 and 2015 from CHARLS. The test group included respondents with healthy kidney function in 2011 and followed them up until 2015. We recruited 1,303 respondents from modeling groups and 1980 respondents from the test group (Supplementary Figure S1). In addition, 1980 individuals were confirmed as having healthy kidney health in 2011 and also followed up to 2015 (Supplementary Figure S2). The flow design of our study is shown in Figure 1.

### 3.2 General characteristics of respondents in different groups

The general characteristics of respondents were described. The median CA of the modeling group in 2011 and 2015 were 56.00 (49.00, 62.00), and 60.00 (53.00, 66.00) respectively. The median CA of the test group in 2011 and 2015 were 57.00 (50.00, 64.00), and 61.00 (54.00, 68.00), respectively (Supplementary Table S1).

### 3.3 The result of the principal component analysis

#### 3.3.1 Determination of variables in the BA of kidney

The data of 1,303 respondents in 2011 were used to calculate the biological age for determining the efficiency of the two methods. Initially, variables associated with kidney health were included in the BA of the kidney based on the strength of their relationship with CA, |*r*| > 0.10 (*p* < 0.05). The variables were eliminated for the collinearity of each other, |*r*| < 0.60 (*p* < 0.05). Only the most correlative one could be remained. Finally, blood urea nitrogen (bun), creatinine (crea), uric acid (ua), and cystatin C (cysc) were included (Supplementary Figure S3).

#### 3.3.2 Construction of the BA of kidney

The selected four biomarkers (bun, crea, ua, and cysc) were further analyzed by PCA to reduce the dimension and identify the most important factors associated with the aging process. Only the eigenvalue of the first principal component was greater than 1.0 (10) (Supplementary Table S2). All the biomarkers *Xn* were transformed into standardized form (*Xn*-*Xmean*)/*SDn*. BAS was calculated as BAS = 0.609*(*X1-crea_mean_*)/*SD_crea_* + 0.558*(*X2-ua_mean_*)/*SD_ua_* + 0.434*(*X3-cysc_mean_*)/*SD_cysc_* + 0.359*(*X4-bun_mean_*)*/SD_bun_*. The *BA* transformed by T-score was used as an intermediate variable to compare with *CA*. To eliminate the edge data distortion of the BA formula, the Z score was added to the equation to correct BA. Eventually, we acquired the biological age by PCA, PCA-BAc.

### 3.4 Klemera and Doubal’s method

The basic parameters of the modeling group in 2011 were calculated as follows. These parameters estimated KD-BA and KD-BAec (Supplementary Table S3).

### 3.5 Assessment of different biological age-estimated methods

We evaluated the two methods using *r* and *r^2^*, as shown in Supplementary Table S4. Firstly, we constructed the biological age formulas with 1,303 respondents of the modeling group. The results showed that PCA-BAc and KD-BAec performed 0.65 and 0.57 of *r*, 0.42 and 0.33 of *r^2^*, respectively. The results showed a stable trend in the two modeling methods (Supplementary Table S4; Supplementary Figure S4). Comparison of PCA-BAc and KD-BAec to CA for the same year showed that the results of the two methods in 2011 had no statistical difference, indicating that the CA of individuals with a healthy kidney could reflect BA. In 2015, the difference between PCA-BAc and CA was −1.84, indicating that the BA was 1.84 years younger than the *CA.* In contrast, the difference between KD-BAec and CA was 8.43, indicating that the biological age was 8.43 years older than the chronological age. We also compared the BA of kidney differences between 2015 and 2011. We found that the difference between the BA of the kidney calculated by the PCA and the CA (ΔPCA-BAc 2015–2011) was 2.88 years old after 4 years of follow-up. The difference between the BA of the kidney calculated by the KD and the CA (ΔKD-BAec 2015–2011) was 12.05 years older (Supplementary Table S5). In contrast to the result of the PCA method, the modeling method of KDM was illogical and ruled it out.

### 3.6 Applying the model for biological age

After verifying the reliability of the model, it was applied to a kidney health cohort for evaluating kidney function. The 1980 respondents of the test group with healthy kidney function calculated the BA of the kidney in 2011. After the follow-up, only 576 respondents had kidney diseases or kidney dysfunction. Then we calculated the BA of 1980 respondents in 2015. The BA of the kidney at baseline had no significant differences from *CA.* The *r* of PCA-BAc was 0.57 (*p* < 0.05), and the *r*^2^ was 0.33 (*p* < 0.05). After a 4-year follow-up, the *r* and *r*^2^ of models increased compared with the baseline (PCA-BAc, *r* = 0.67, *r*^2^ = 0.44) (Supplementary Figure S5). The difference between PCA-BAc from 2011 to 2015 was 2.67 years (Supplementary Tables S6, S7).

#### 3.6.1 The BA of kidney and kidney diseases

After follow-up of the test group, 48 of 1980 respondents (2.4%) were considered as having kidney disease or kidney dysfunction, diagnosed by the level of eGFR <60 mL/min/1.73m^2^ or a history of kidney disease, who had healthy kidney function at baseline.

**Figure 2:**
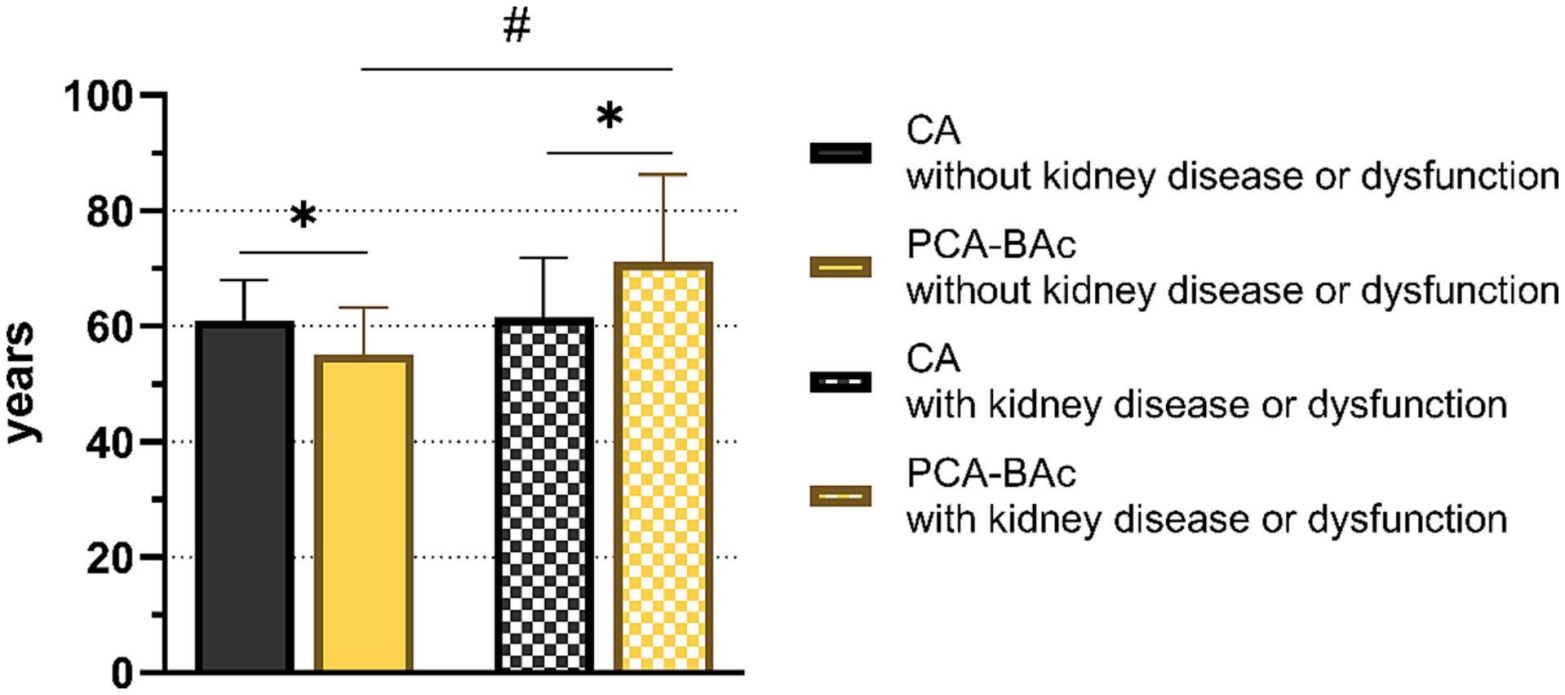
The biological age and chronological age of respondents with and without kidney diseases or dysfunction.

The BA of the kidney appeared different in the two outcomes after 4 years of follow-up, PCA-BAc of respondents with kidney disease or kidney dysfunction (PCA-BAc = 71.20, *p* < 0.05) and those without kidney disease or kidney dysfunction (PCA-BAc = 55.11, *p* < 0.05). However, CA was not significantly different (with PCA-BAc = 61.50; without PCA-BAc = 61.00, *p* > 0.05). There was no statistical difference between PCA-BAc and CA of respondents without kidney disease or kidney dysfunction during follow-up (PCA-BAc = 55.11, CA = 61.0, *p* = 0.300), and they were younger than their chronologic age. However, there was a significant statistical difference between PCA-BAc and CA of respondents with kidney disease or kidney dysfunction after 4 years of follow-up (PCA-BAc = 71.20, CA = 61.50, *p* < 0.05), and the BA was older than the *CA.* Comparing the difference in biological age between 2015 and 2011 (ΔPCA-BAc 2015–2011), it was found that respondents without kidney disease or kidney dysfunction were 2.83 years younger than *CA.* In contrast, respondents with kidney diseases or kidney dysfunction were 6.10 years older than the CA average (Figure 2). The BA of kidney performed better than CA in distinguishing people with or without kidney disease or kidney dysfunction.

#### 3.6.2 The BA of the kidney and the different levels of eGFR

**Figure 3:**
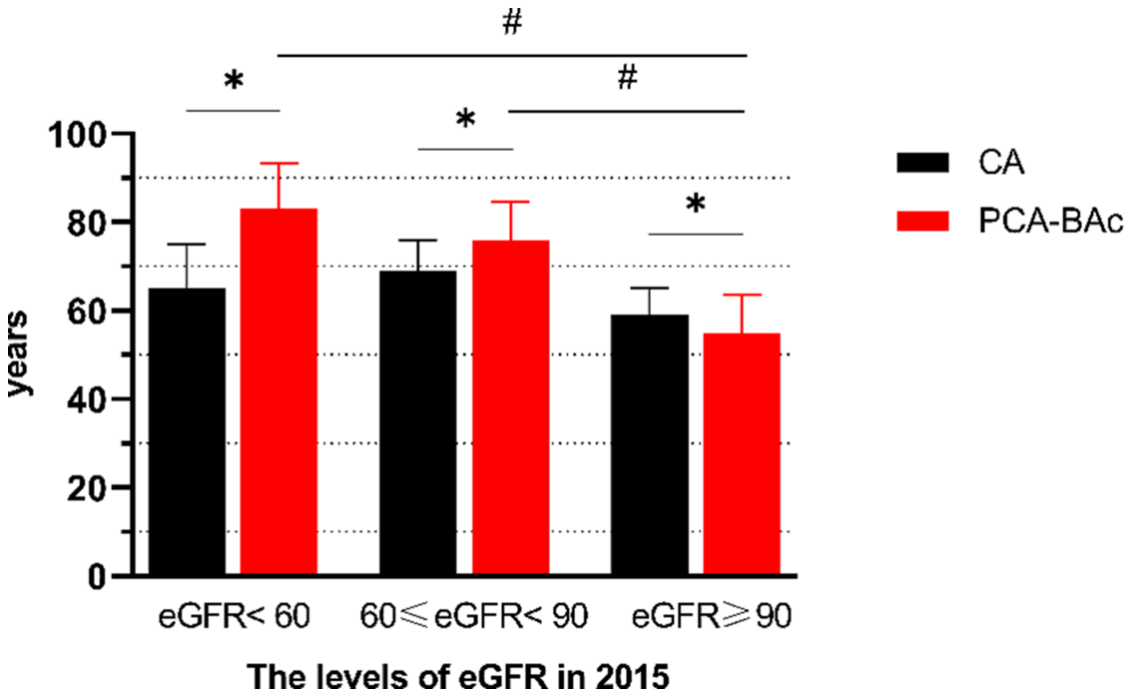
The biological age and chronological age of respondents with different kidney functions.

To further understand the BA of respondents with different kidney function levels, we divided the respondents of the test group in 2015 into eGFR <60, 60 ≤ eGFR <90, and eGFR ≥90 groups according to the standard of kidney disease classification. CA and BA of every kidney level group were different significantly (eGFR <60: CA = 65.0, PCA-BAc = 83.2; 60 ≤ eGFR <90: CA = 69.0, PCA-BAc = 75.9; eGFR ≥90:CA = 59.0, PCA-BAc = 54.8). The BA of eGFR <60 group and 60 ≤ eGFR <90 were older than *CA.* The BA of eGFR ≥90 groups were younger than *CA.* The differences between BA and CA from big to small were eGFR <60, 60 ≤ eGFR <90, and eGFR ≥90. The oldest BA of the kidney was the eGFR <60 group. The difference of PCA-BAc in the following 4 years (ΔPCA-BAc 2015–2011) was 12.14 years old (eGFR <60), 3.91 years old (60 ≤ eGFR <90), and 2.16 years old (eGFR ≥90), respectively. All the above results showed that CA and PCA-BAc increased as kidney function decreased, especially PCA-BAc (Supplementary Table S8; Figure 3).

#### 3.6.3 The BA of the kidney and the different levels of the kidney at baseline

**Figure 4:**
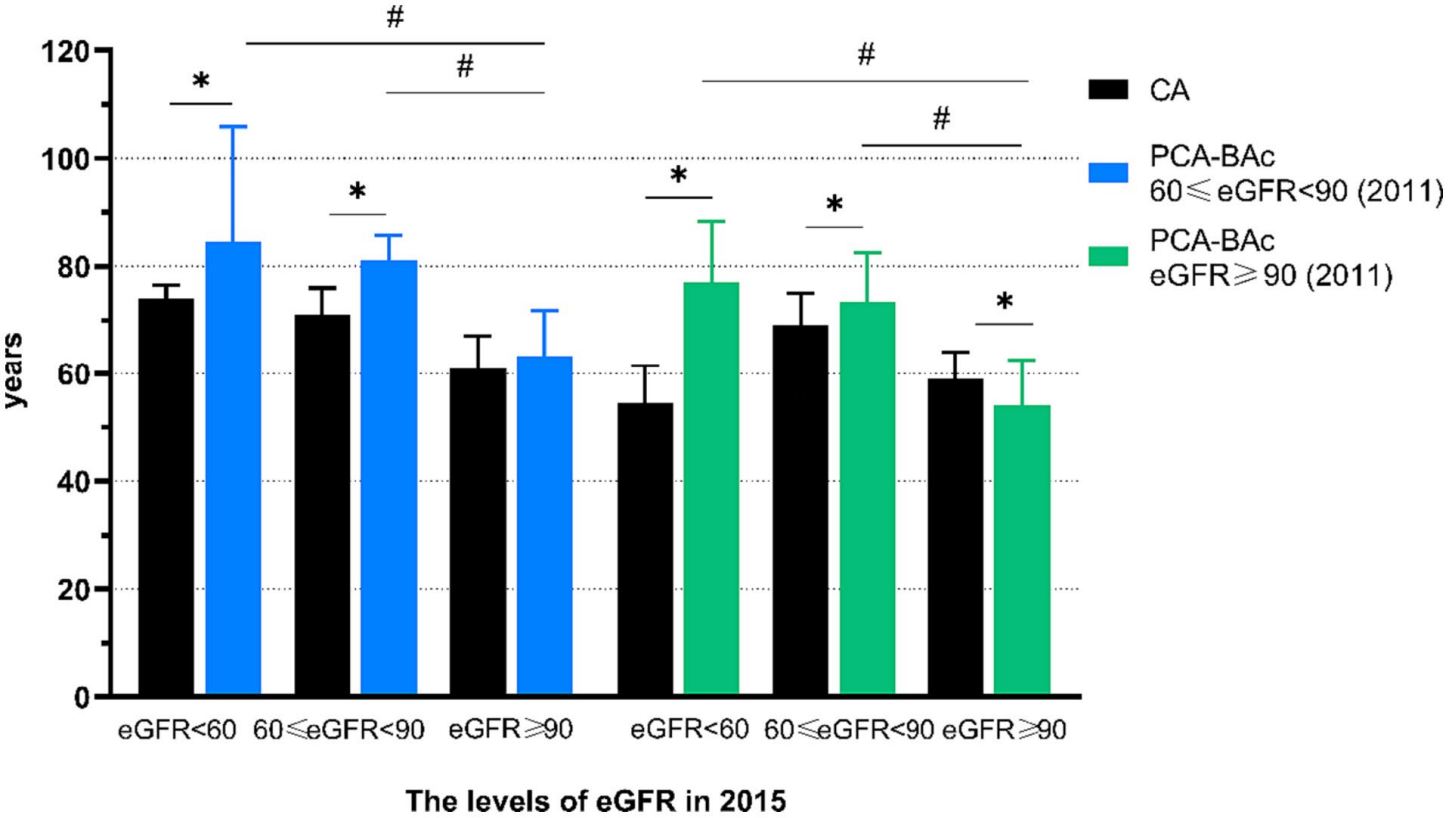
The biological age and chronological age of respondents with different kidney function with the different level at baseline.

The healthy kidney respondents had different outcomes after a 4-year follow-up. We aimed to determine whether there were differences in the outcomes of individuals with different levels of eGFR at the baseline. Therefore, we divided the healthy kidney respondents into two groups, 60 ≤ eGFR <90 and eGFR ≥90 at baseline and compared the outcomes. We found 399 respondents with 60 ≤ eGFR <90 and 1,580 respondents with eGFR ≥90 at baseline. Then we tracked the outcomes of two groups. We found BA of the 60 ≤ eGFR <90 group was older than the eGFR ≥90 group after 4 years of follow-up. Then we divided kidney function into 3 outcomes at the follow-up year. We found that the BA of 60 ≤ eGFR <90 and eGFR <60 differed from those of eGFR ≥90, even though these three groups began at the same baseline level (Supplementary Table S8; Figure 4).

#### 3.6.4 Association of BA of the kidney with the risk of decreased kidney function

To further understand the risk of different kidney levels at baseline or after follow-up, we applied multinomial logistic regression. Among respondents with 60 ≤ eGFR <90 at baseline, the risk of those with eGFR <60 after follow-up increased by 41.5% for each additional year of PCA-BAc compared with eGFR ≥90, and 60 ≤ eGFR <90 after follow-up increased by 21.3%. Similarly, the risk of those with eGFR <60 after follow-up increased by 21.9% for each additional year of PCA-BAc difference in 4 follow-up years compared with those with eGFR ≥90, and 60 ≤ eGFR <90 after follow-up increased by 9.8%. The risk of those with eGFR <60 in 2015 increased by 13.2% for each additional year of CA compared with eGFR ≥90, and 60 ≤ eGFR <90 increased by 11.1%. All the above results showed BA of the kidney performed an obvious difference than CA for evaluating risks of kidney function decreased.

**Figure 5:**
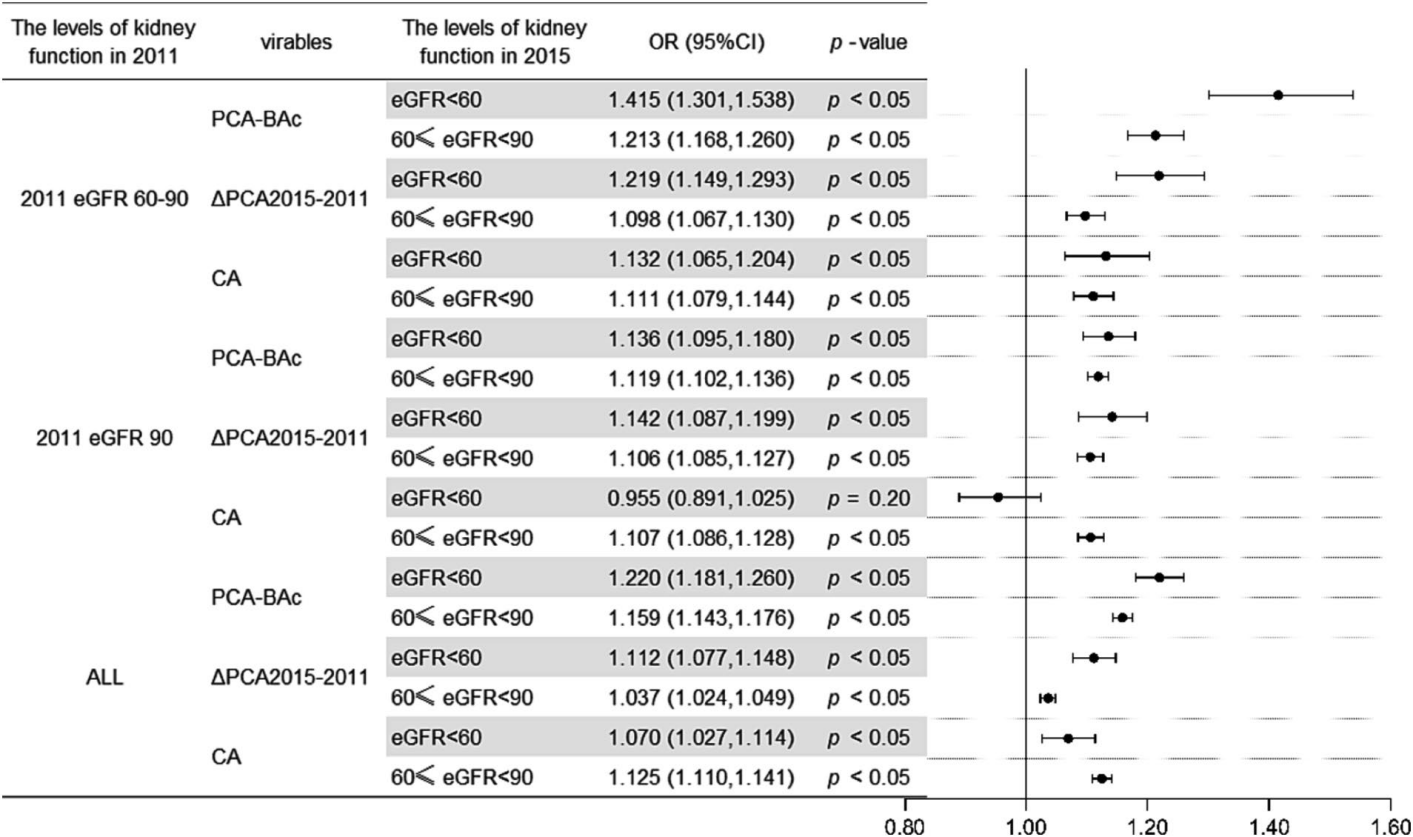
Odds ratios plot of different levels of eGFR in 2015 by kidney functions on the baseline.

Among respondents with eGFR ≥90 at baseline, the risk of those with eGFR <60 after follow-up increased by 13.6% for each additional year of PCA-BAc compared with eGFR ≥90, and 60 ≤ eGFR <90 increased by 11.9%. The risk of those with eGFR <60 after follow-up increased by 14.2% for each additional year of PCA-BAc difference in 4 followed-up years compared with eGFR ≥90, and 60 ≤ eGFR <90 after follow-up increased by 10.6%. In the same way, the risk of those with eGFR <60 after follow-up decreased insignificantly by 4.5% for each additional year of CA compared with eGFR ≥90, and 60 ≤ eGFR <90 after follow-up increased by 10.7%. The results of all respondents were in Supplementary Table S9 and Figure 5. The results above indicated that the kidney function after follow-up was correlated with BA and *CA.* The healthier kidney function at baseline, the less kidney function decreased after follow-up, and the less risk induced by PCA-BAc.

## 4 Discussion

In our study, we aimed to build a model of kidney biological age for evaluating the kidney function of individuals. Therefore, we screen a population with a healthy kidney from CHARLS and regard their CA as BA of kidney for modeling. The models were constructed by two mainstream methods, PCA and KDM. Selected from statistics and logic step by step, the model of KDM was eliminated for illogical and PCA-BAc remained for further analysis. Our purpose of the model was to evaluate the kidney function more elaborated than eGFR among those with or without kidney diseases. Therefore, we tracked a group of test population with healthy kidneys from CHARLS at baseline and followed them up after 4 years, comparing the differences of BA in multi-dimensions among various levels of kidney function.

Firstly, the BA of the test group was 1.33 years younger than their *CA.* The whole test group at baseline had normal kidney function. After follow-up, 48 (2.4%) respondents had kidney diseases or dysfunction. PCA-BAc was significantly different between respondents with and without kidney disease or dysfunction, but not *CA.* It was suggested that PCA-BAc, composed of kidney biomarkers, can reflect kidney function more accurately than *CA.* Researchers found that biomarkers greater than the cutoff values indicated an increase in acute kidney injury risk, irrespective of kidney function (21). Cystatin C is an independent predictor of all-cause mortality among middle-aged and older adults in Chinese (22). Quantification of biological aging among Taiwanese older adults, a traditional biomarker index was performed as well as participant self-rated health to predict these outcomes (23). In the Framingham offspring study, the BA of the kidney was regarded as complementary in predicting risk for mortality and age-related diseases (24). BA of the kidney was better than CA for measuring life and health span in the Singapore Longitudinal Aging Study (25). The research above expressed our point again. We found the difference of PCA-BAc during 4 years of follow-up was very close to 4 years, when individuals merely maintained 60 ≤ eGFR <90.

Secondly, the respondents with healthy kidneys had different outcomes after a 4-year follow-up. We investigated whether there were differences in outcomes between individuals with different levels of eGFR at the baseline. We divided respondents into the normal kidney function group (60 ≤ eGFR <90) and the good kidney function group (eGFR >90). Regardless of kidney condition, PCA-BAc increased as kidney function decreased. The PCA-BAc of respondents with good kidney conditions was younger than those with a normal condition. In other words, keeping a good kidney function at the baseline was beneficial to obtain a better outcome in the future.

Thirdly, it was known that kidney function decreased with aging even in healthy populations. Some studies have reported that eGFR decreased with aging (26, 27). or in diseases (17, 21, 22, 24, 28, 29) Comparing the effect of indicators was meaningful. Accordingly, using the ratios of different levels of eGFR after follow-up by kidney functions compared with the reference group, we determined the risk of those whose kidney function was eGFR <60 and 60 ≤ eGFR <90 increased significantly for each additional year of PCA-BAc compared with eGFR ≥90. The group with higher levels of kidney function had a lower risk. The individuals with a higher level of kidney function at the baseline had a lower risk for every additional year of PCA-BAc. Norwegian Patients with IgA Nephropathy reported that the risk of end-stage renal disease or deaths of patients with 60 ≤ eGFR and 30 ≤ eGFR <59.9 are 5.7 and 18.7 times higher than patients with eGFR ≥60 (30). Moreover, the risk was more evident in the PCA-BAc than the CA, confirming the superiority of biological age in estimating aging.

eGFR was a classic and wide standard for evaluating kidney function. However, its accurate grouping was focused on kidney disease not healthy people. It was a blank for people in the middle of the health and diseases. The typical characteristics of kidney diseases were less awareness and strong stealthiness. Kidney biological age was an appropriate tool for evaluating comprehensively and conveniently. The plain results gave the public a direct report and comparison, which improved the awareness effectively. That was the core of our study. However, this was a study that preliminarily explored the biological age model in evaluating kidney function based on an online database. One limitation was the lack of kidney function biomarkers collected by CHARLS so that less representativeness of biomarkers. Another limitation was that we did not account for the kidney’s endocrine system. The relationship between RAAS and senescence, and RAAS hyperactivity, appeared to be one of the primary inducing mechanisms for normal senescence and many prevalent diseases in older adults (31). A collection of biomarkers was the basis for a comprehensive evaluation of kidney function. We suggest that the above reasons caused KDM unsuitable for our research. It could involve the application of statistics.

## 5 Conclusion

In conclusion, BA of the kidney is a more precise parameter for estimating aging than CA and correlates negatively with reduced kidney function. The individual with a better kidney condition means a younger BA of kidney and a better outcome in the following years. The model of kidney biological age fills the blank of evaluation among people in the middle of the heathy and kidney diseases.

## Data availability statement

The original contributions presented in the study are included in the article/Supplementary material, further inquiries can be directed to the corresponding author.

## Ethics statement

The studies involving humans were approved by the Ethics Review Committee of Peking University (IRB 00001052-11015). The studies were conducted in accordance with the local legislation and institutional requirements. Written informed consent for participation in this study was provided by the participants’ legal guardians/next of kin.

## Author contributions

SP: Data curation, Formal analysis, Software, Visualization, Writing – original draft. RX: Investigation, Writing – review & editing. KW: Software, Validation, Writing – review & editing. NL: Software, Writing – review & editing. YuL: Supervision, Writing – original draft. YoL: Conceptualization, Funding acquisition, Methodology, Writing – original draft.

## Footnotes

^1^http://charls.pku.edu.cn/index/zh-cn.html

^2^https://cran.r-project.org
