# Supplementary material for: Association between kidney function and biological age: a China Health and Retirement Longitudinal Study
Source: Front Public Health. 2023 Dec 18;11:1259074. doi: 10.3389/fpubh.2023.1259074 (PMC10757928; doi:10.3389/fpubh.2023.1259074)
Supplement: Supplementary file 1 [file Table_1.DOCX]

**Supplementary Materials (Figures)**


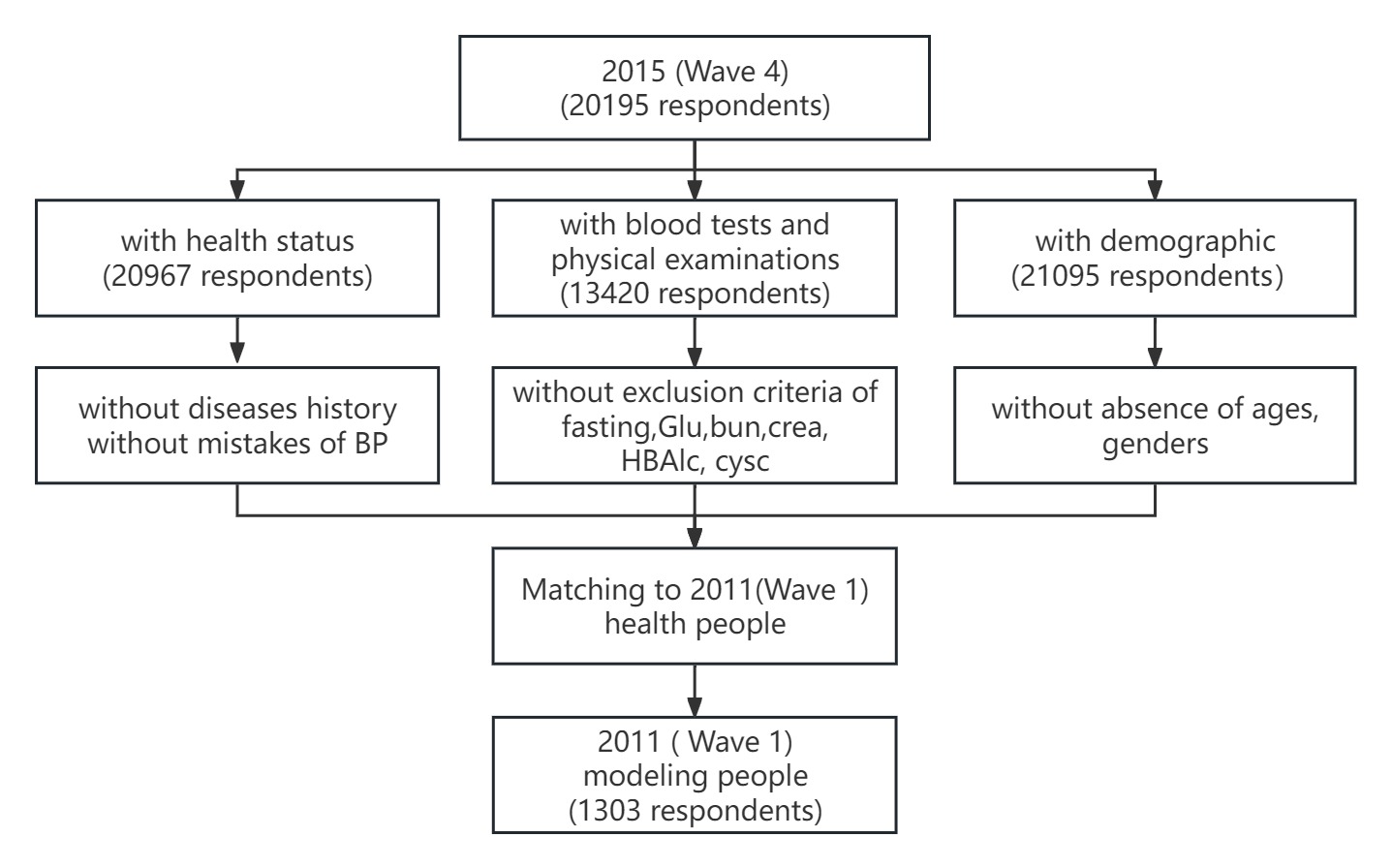
**Article title:** Association Between Kidney Function and Biological Age: A China Health and Retirement longitudinal study (CHARLS)

**Supplementary Fig. S1.** The inclusion criteria of respondents of modeling group


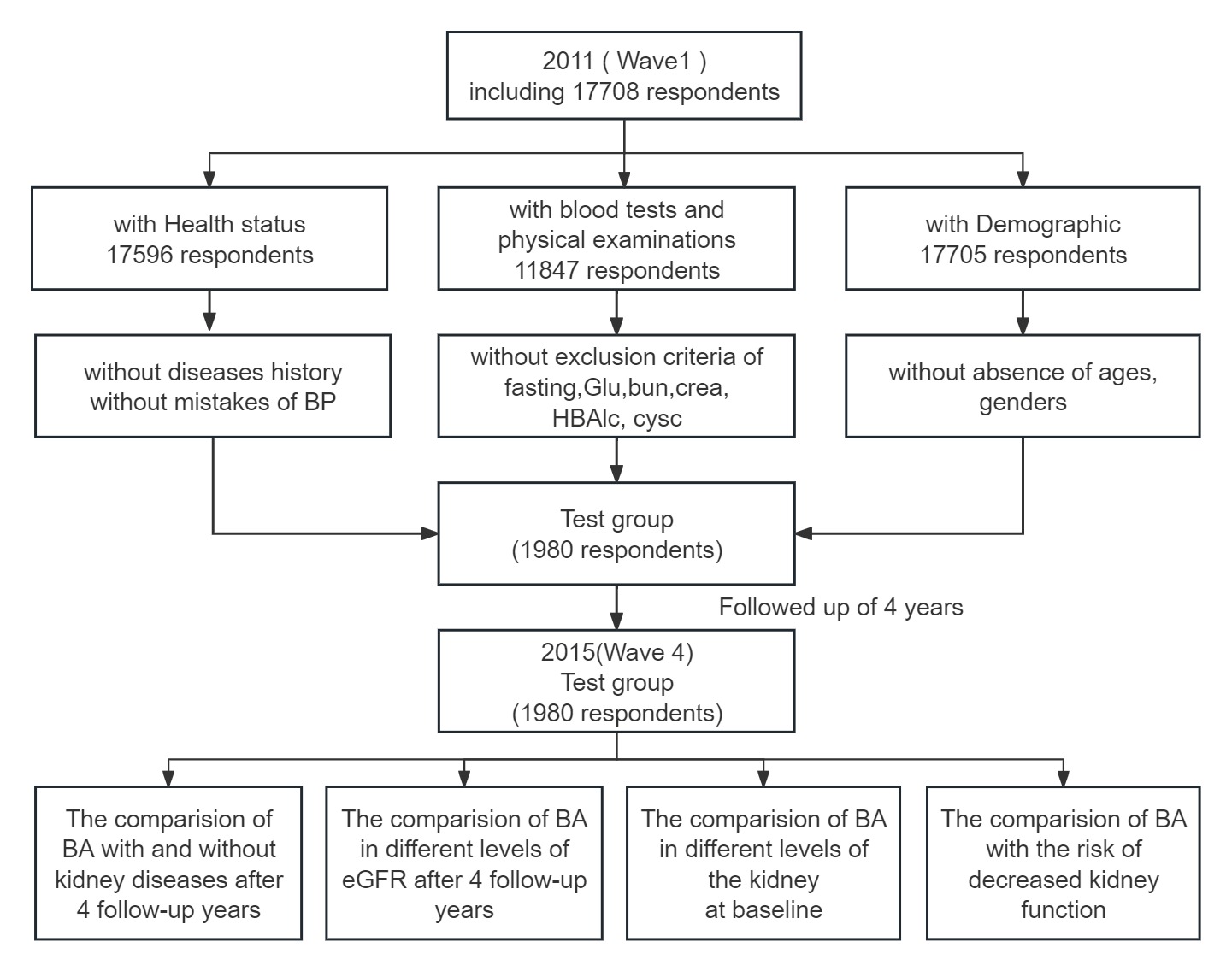


**Supplementary Fig. S2.** The inclusion criteria of respondents of Test group


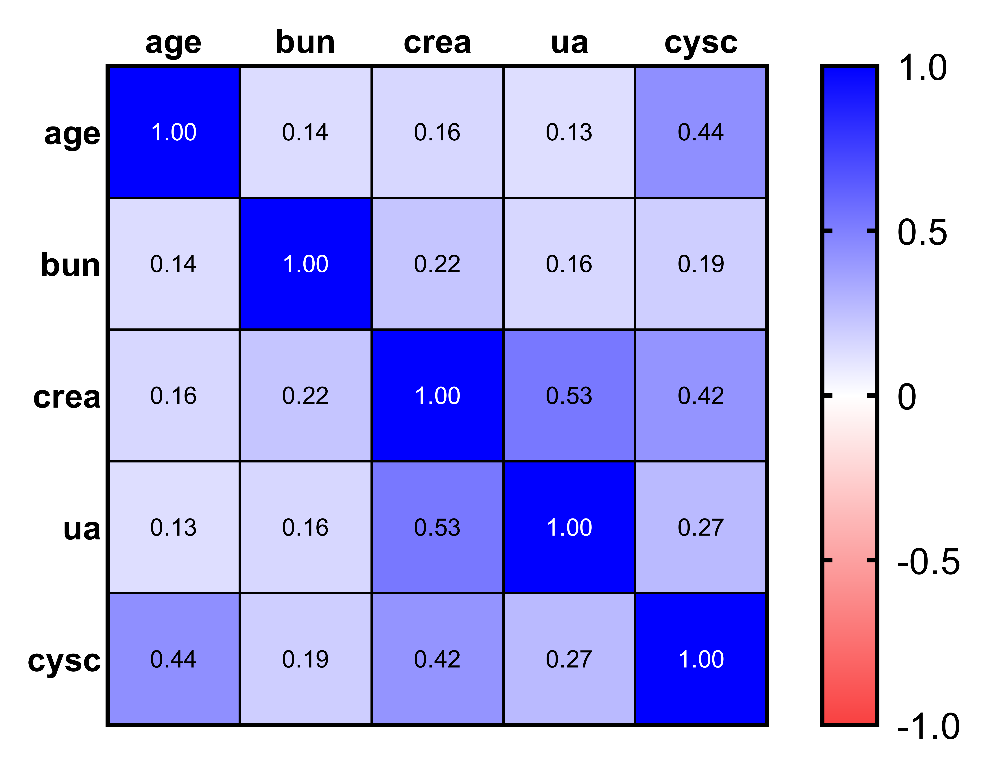


**Supplementary Fig. S3.** The correlation of kidney biomarkers


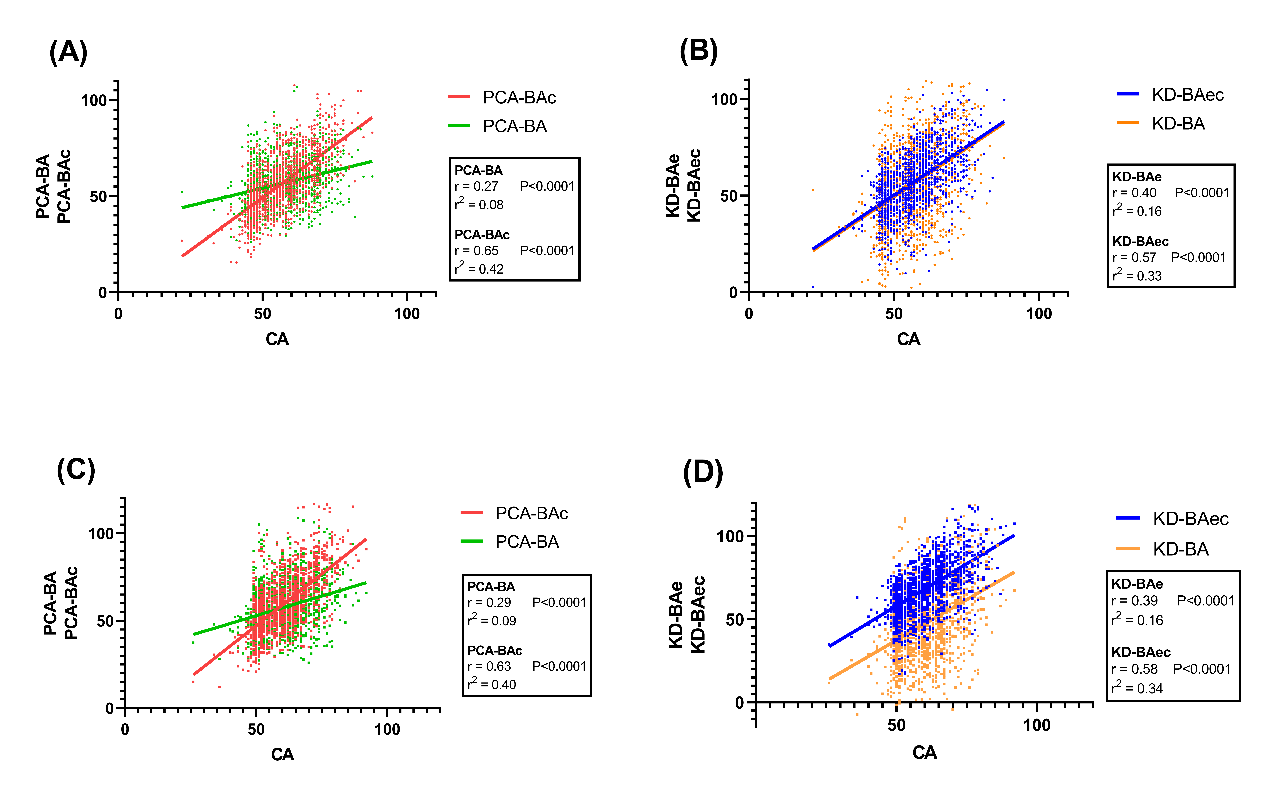


**Supplementary Fig. S4.** The correlation coefficient and the fitting degree of PCA and KDM

The r and r2 of PCA-BA and PCA-BAc of the modeling group at baseline (A), KD-BA and KD-BAec of the modeling group at baseline (B), PCA-BA and PCA-BAc of the modeling group after follow-up (C), KD-BA and KD-BAec of modeling group after follow-up(D)


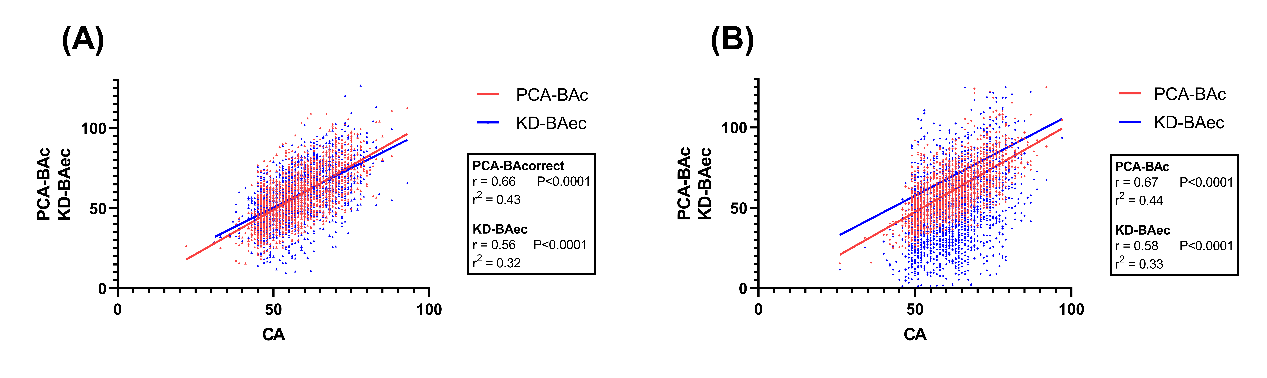


**Supplementary Fig. S5.** The correlation coefficient and the fitting degree of PCA and KDM of the test group (at baseline (left), after follow-up (right))
