# Supplementary material for: Association between kidney function and biological age: a China Health and Retirement Longitudinal Study
Source: Front Public Health. 2023 Dec 18;11:1259074. doi: 10.3389/fpubh.2023.1259074 (PMC10757928; doi:10.3389/fpubh.2023.1259074)
Supplement: Supplementary file 2 [file Table_2.DOCX]

**Supplementary material （Tables）**

**Article title:** Association Between Kidney Function and Biological Age: A China Health and Retirement longitudinal study (CHARLS)

**Supplementary Table S1.** Characteristics of Respondents in Different Groups

| Characteristics | Modeling group | | Test group | |
| --- | --- | --- | --- | --- |
|  | Baseline (n=1303) | Follow-up (n=1303) | Baseline (n=1980) | Follow-up (n=1980) |
| age | 56.00 (49.00, 62.00) | 60.00 (53.00, 66.00) | 57.00 (50.00, 64.00) | 61.00 (54.00, 68.00) |
| gender |  |  |  |  |
| male | 611 (46.90%) | 607 (46.58%) | 919 (46.41%) | 919 (46.41%) |
| female | 692 (53.07%) | 696 (53.42%) | 1061 (53.59%) | 1061 (53.59%) |
| bun | 15.24 (12.69, 17.98) | 14.85 (12.32, 18.21) | 15.15 (12.63, 18.03) | 14.85 (12.32, 18.21) |
| crea | 0.73 (0.64, 0.85) | 0.75 (0.66, 0.88) | 0.73 (0.63, 0.85) | 0.75 (0.66, 0.88) |
| ua | 4.03 (3.39, 4.80) | 4.60 (3.80, 5.50) | 4.07 (3.42, 4.88) | 4.60 (3.80, 5.60) |
| cysc | 0.95 (0.84, 1.07) | 0.81 (0.71, 0.93) | 0.95 (0.84, 1.08) | 0.82 (0.72, 0.94) |

**Supplementary Table S2.** The Eigenvalues and Explained Variances of Components

|  | Component 1 | Component 2 | Component 3 | Component 4 |
| --- | --- | --- | --- | --- |
| crea | 0.824 | - | - | - |
| ua | 0.755 | - | - | - |
| cysc | 0.588 | - | - | - |
| bun | 0.486 | - | - | - |
| Eigenvalues | 1.937 | 0.892 | 0.732 | 0.438 |
| Variance, % | 48.429 | 22.300 | 18.312 | 10.959 |
| Cumulative Variance % | 48.429 | 70.729 | 89.041 | 100.000 |

**Supplementary Table S3.** The Parameters of Klemera and Doubal Method Biological Age Algorithm

|  | Intercept | slope | correlation coefficient | root mean square error |  |  |  |  |
| --- | --- | --- | --- | --- | --- | --- | --- | --- |
|  | $q$ | $k$ | $r_{\text{ }}$ | $s_{j}^{2}$ | $r_{char\text{ }}$ | $s_{BA}^{2}$ | ${CA}_{max}$ | ${CA}_{min}$ |
| bun | 12.02 | 0.65 | 0.14 | 79.31 |  |  |  |  |
| crea | 0.60 | 0.00 | 0.15 | 0.11 |  |  |  |  |
| ua | 3.25 | 0.02 | 0.13 | 5.40 |  |  |  |  |
| cysc | 0.46 | 0.01 | 0.41 | 0.15 |  |  |  |  |
| Summary Parameters |  |  |  |  | 0.28 | -544.02 | 88.00 | 22.00 |

**Supplementary Table S4.** The Correlation Coefficient and the Fitting Degree of PCA and KDM in Modeling Group

|  | CA | PCA-BAc | KD-BAec |
| --- | --- | --- | --- |
| Modeling group in 2011 (n = 1303) | | | |
| description | 56.00 (49.00,62.00) | 55.71 (46.35,65.91) | 56.96 (46.86,67.58) |
| *r* | 1.00 | 0.65 | 0.57 |
| *r^2^* | 1.00 | 0.42 | 0.33 |
| Modeling group in 2015 (n = 1303) | | | |
| description | 60.00 (53.00,66.00) | 58.22 (48.26,70.39) * | 68.90 (57.91,79.04) * |
| *r* | 1.00 | 0.63 | 0.58 |
| *r^2^* | 1.00 | 0.40 | 0.34 |

* a significant difference existed compared with the CA

**Supplementary Table S5.** The Difference between BA and CA of Modeling Group at Baseline (2011) and Follow-up (2015)

|  | PCA-BAc - CA | KD-BAec - CA | ΔPCA-BAc 2015-2011 | ΔKD-BAec 2015-2011 |
| --- | --- | --- | --- | --- |
| Baseline (2011) | -0.85 (-7.88,7.16) | 0.65 (-7.98,9.32) | 2.88 (-3.01,9.05) | 12.05 (5.19,18.59) |
| Follow-up (2015) | -1.84 (-9.54,7.21) | 8.43 (0.17,16.74) |  |  |

**Supplementary Table S6.** The Correlation Coefficient and the Fitting Degree of PCA and KDM of Test group at Baseline (2011) and Follow-up (2015)

|  | CA | PCA-BAc | KD-BAec |
| --- | --- | --- | --- |
| Test group at baseline in 2011 （n=1980） | |  | |
| description | 57.00 (50.00,64.00) | 56.08 (46.60,66.98) | 57.57 (47.48,69.00) |
| *r* | 1.00 | 0.57 | 0.56 |
| *r^2^* | 1.00 | 0.33 | 0.32 |
| Test group after follow-up in 2015 (n=1980） | | 1980 | |
| description | 61.00 (54.00,68.00) | 58.55 (49.30,69.58) * | 69.14 (58.42,79.95) * |
| *r* | 1.00 | 0.67 | 0.58 |
| *r^2^* | 1.00 | 0.44 | 0.33 |

* a significant difference existed compared with the CA.

**Supplementary Table S7.** The Difference between BA and CA of Test group at Baseline (2011) and Follow-up (2015)

|  | ΔPCA-BAc - CA | ΔKD-BAec - CA | ΔPCA-BAc 2015-2011 | ΔKD-BAec 2015-2011 |
| --- | --- | --- | --- | --- |
| Baseline (2011) | -1.07 (-8.29,6.84) | 0.80 (-7.93,9.46) | 2.67 (-2.98,8.19) | 11.88 (4.50,18.87) |
| Follow-up (2015) | -2.66 (-9.30,5.33) | 8.43 (-0.04,16.94) |  |  |

**Supplementary Table S8.** The BA of Different Kidney Functions Groups

|  | CA | PCA-BAc | ΔPCA2015-2011 |
| --- | --- | --- | --- |
| **with or without kidney diseases or kidney dysfunction in 2015 (n=1932)** | | | |
| **With** (n=48) | 61.50 (56.00,71.75) | 71.20 (61.16,86.20) *# | 6.10 (2.86,13.25) |
| **Without** (n=1932) | 61.00 (54.00,68.00) | 55.11 (48.08,63.15) * | -2.83 (-9.42,4.92) |
| **different levels of eGFR in 2015 (n=48)** | | | |
| **eGFR<60** (n=31) | 65.00 (56.00,75.00) # | 83.20 (75.49,93.21) *# | 12.14 (7.67,17.52) |
| **60≤eGFR<90** (n=449) | 69.00 (62.00,76.00) # | 75.91 (64.16,84.50) *# | 3.91 (-2.04,10.97) |
| **eGFR≥90** (n=1500) | 59.00 (53.00,65.00) | 54.82 (46.84,63.48) * | 2.16 (-3.39,7.28) |
| **The eGFR in 2015 of respondent with 60≤eGFR<90 at baseline （n=399）** | | | |
| **eGFR<60** (n=16) | 74.0 (66.0,76.5) # | 84.53 (81.17,105.80) *# | 9.84 (6.42,16.27) |
| **60≤eGFR<90** (n=255) | 71.0 (64.0,76.0) # | 81.11 (75.40,85.74) *# | 0.70 (-4.72,6.08) |
| **eGFR≥90** (n=128) | 61.0 (58.0,67.0) | 63.27 (55.58,71.76) | -5.76 (-10.59, -1.17) |
| **The eGFR in 2015 of respondent with eGFR≥90 at baseline （n=1580）** | | | |
| **eGFR<60** (n=14) | 54.50 (49.75,61.50) | 76.96 (62.98,88.23) *# | 13.97 (9.88,17.62) |
| **60≤eGFR<90** (n=194) | 69.00 (58.00,75.00) # | 73.47 (61.76,82.57) *# | 8.81 (2.81,14.94) |
| **eGFR≥90** (n=1372) | 59.00 (52.00,64.00) | 54.04 (46.25,62.48) * | 2.79 (-2.28,7.67) |

* a significant difference existed compared with CA of the same year.

# a significant difference existed compared with the kidney function of eGFR≥90

**Supplementary Table S9.** Odds Ratios (ORs) and 95% Confidence Intervals (CIs)of Different Levels of eGFR in 2015 by Kidney Functions on the Baseline Compared with the Reference Group (eGFR ≥90 in 2015) Using the Multinomial Logistic Regression Model

| The levels of eGFR in 2015 | eGFR<60 | | 60≤eGFR<90 | |
| --- | --- | --- | --- | --- |
|  | OR (95%CI) | P | OR (95%CI) | P |
| 2011 60≤eGFR<90 |  |  |  |  |
| PCA-BAc | 1.415 (1.301,1.538) | <0.05 | 1.213 (1.168,1.260) | <0.05 |
| ΔPCA-BAc 2015-2011 | 1.219 (1.149,1.293) | <0.05 | 1.098 (1.067,1.130) | <0.05 |
| CA | 1.132 (1.065,1.204) | <0.05 | 1.111 (1.079,1.144) | <0.05 |
| 2011 eGFR≥90 |  |  |  |  |
| PCA-BAc | 1.136 (1.095,1.180) | <0.05 | 1.119 (1.102,1.136) | <0.05 |
| ΔPCA-BAc 2015-2011 | 1.142 (1.087,1.199) | <0.05 | 1.106 (1.085,1.127) | <0.05 |
| CA | 0.955 (0.891,1.025) | 0.2 | 1.107 (1.086,1.128) | <0.05 |
| ALL |  |  |  |  |
| PCA-BAc | 1.220 (1.181,1.260) | <0.05 | 1.159 (1.143,1.176) | <0.05 |
| ΔPCA-BAc 2015-2011 | 1.112 (1.077,1.148) | <0.05 | 1.037 (1.024,1.049) | <0.05 |
| CA | 1.070 (1.027,1.114) | <0.05 | 1.125 (1.110,1.141) | <0.05 |

**Supplementary Table S10.** Abbreviation

| China Health and Retirement Longitudinal Study | CHARLS |
| --- | --- |
| chronological age | CA |
| biological age | BA |
| principal component analysis | PCA |
| Klemera and Doubal's method | KDM |
| estimated glomerular filtration rate | eGFR |
| fasting blood glucose | glu |
| non-fasting blood glucose | uglu |
| glycated hemoglobin | HbA1c |
| uric acid | ua |
| creatinine | crea |
| urea nitrogen | bun |
| cystatin C | cysc |
| biological age score | BAS |
| Kidney biological age modeling by PCA | PCA-BAc |
| Kidney biological age modeling by KDM | KD-BAec |
